# Immunoaffinity Cleanup and Isotope Dilution-Based Liquid Chromatography Tandem Mass Spectrometry for the Determination of Six Major Mycotoxins in Feed and Feedstuff

**DOI:** 10.3390/toxins14090631

**Published:** 2022-09-11

**Authors:** Ying Liu, Yongpeng Jin, Qi Guo, Xiong Wang, Sunlin Luo, Wenjun Yang, Juntao Li, Yiqiang Chen

**Affiliations:** 1 State Key Laboratory of Animal Nutrition, College of Animal Science and Technology, China Agricultural University, Beijing 100193, China; 2 Clover Technology Group Inc., Beijing 100044, China

**Keywords:** LC-MS/MS, mycotoxins, feed, feedstuff, immunoaffinity column, isotope internal standard

## Abstract

In this study, a liquid chromatography-tandem mass spectrometry (LC-MS/MS) method for simultaneous determination of deoxynivalenol, aflatoxin B_1_, zearalenone, ochratoxin A, T-2 toxin and fumonisin B_1_ in feed and feedstuff was established. The sample was extracted with an acetonitrile–water mixture (60:40, *v*/*v*), purified by an immunoaffinity column, eluted with a methanol–acetic acid mixture (98:2, *v*/*v*), and reconstituted with a methanol–water mixture (50:50, *v*/*v*) after drying with nitrogen. Finally, the reconstituted solution was detected by LC-MS/MS and quantified by isotope internal standard method. The six mycotoxins had a good linear relationship in a certain concentration range, the correlation coefficients were all greater than 0.99, the limits of detection were between 0.075 and 1.5 µg·kg^−1^, and the limits of quantification were between 0.5 and 5 µg·kg^−1^. The average spike recoveries in the four feed matrices ranged from 84.2% to 117.1% with relative standard deviations less than 11.6%. Thirty-six actual feed samples were analyzed for mycotoxins, and at least one mycotoxin was detected in each sample. The proposed method is reliable and suitable for detecting common mycotoxins in feed samples.

## 1. Introduction

Mycotoxins are biologically active secondary metabolites produced by various fungi, such as *Aspergillus*, *Penicillium* and *Fusarium*. Mycotoxins are widely present in various food and feed [1]. More than 300 mycotoxins have been identified, the most common of which are aflatoxin B_1_ (AFB_1_), deoxynivalenol (DON), zearalenone (ZEN), ochratoxin A (OTA), T-2 toxin (T-2) and fumonisin B_1_ (FB_1_) [2]. Every year, about 25% of crops in the world are contaminated with mycotoxins, causing huge economic losses to the livestock industry [3,4]. Studies have shown that mycotoxins can lead to immunosuppression, and also have a series of potential toxic effects such as hepatotoxicity, nephrotoxicity, immunotoxicity, carcinogenic teratogenicity, and estrogen-like effects [5,6,7,8,9]. On farms, chronic exposure to mycotoxins for animals will result in reduced feed intake [10], reduced feed conversion efficiency [11], increased morbidity [12], reproductive performance degradation [13,14], etc. In addition, mycotoxins in feed can also accumulate in animal-derived food such as meat, eggs, and milk, and thus may pose potential hazards to human health [15]. Economic losses and health risks from mycotoxins in feed have become a global concern. Currently, some regulations have been established for the limit of six major mycotoxins in feed and feedstuff to decrease their toxicological effects in farm animals in many countries. In the European Union, strict limits for mycotoxins in feed such as pig compound feed were regulated in the instruction of 2006/576/EC with limit values of 0.01, 0.9, 0.25, 0.05 and 5 mg·kg^−1^ for AFB_1_, DON, ZEN, OTA, and Fumonisins (B_1_ + B_2_), respectively [16]. Maximum tolerated levels of 0.01, 1.0, 0.25, 0.1, 0.5 and 5 mg·kg^−1^ for AFB_1_, DON, ZEN, OTA, T-2 toxin and Fumonisins (B_1_ + B_2_) in pig compound feed were also set in China [17]. Taken together, it is of great importance to monitor the concentrations of mycotoxins in feed and feedstuff.

The detection methods of mycotoxins mainly include thin layer chromatography (TLC) [18], enzyme-linked immunosorbent assay (ELISA) [19,20], high performance liquid chromatography (HPLC) [20,21,22] and liquid chromatography–tandem mass spectrometry (LC-MS/MS) [23,24]. Among them, the LC-MS/MS method has high accuracy, and it is widely used in the quantitative analysis of mycotoxins in feed. During the extraction process, the fat, protein, pigment and other substances present in feed will also be extracted at the same time, which will interfere with the analysis. It is therefore necessary to purify the sample to remove these impurities. The purification methods of various mycotoxin extracts in feed include QuEChERS (quick, easy, cheap, effective, rugged, and safe) method, multifunctional purification column and immunoaffinity column (IAC), among which IAC achieves a better purification performance [25,26,27]. The principle of the IAC purification method is based on the specific binding of antibodies and antigens, and the purification effect is excellent, which can ensure a good recovery ratio. IAC has been successfully applied to the determination of mycotoxins in feed and feedstuff by LC-MS/MS, but mostly single mycotoxin. Li et al. [28] developed a DON monoclonal antibody-based IAC as a purification tool, and successfully determined the DON content in cereals by ultra-high performance liquid chromatography–tandem mass spectrometry (UPLC-MS/MS). AlFaris et al. [29] applied IAC to the determination of regulated aflatoxins in baby food and feeds and performed satisfactory recoveries. Surely, multifunctional IACs for two or three kinds of mycotoxins were certain reported. In the research of Li et al., a multiple IAC cleanup-based LC-MS/MS method for monitoring DON and T-2 toxin in cereal samples were developed [30]. However, IACs for more than three mycotoxins were rarely reported. Although a report of McKay et al. achieved the analysis of 11 mycotoxins in animal feed by LC-MS/MS with a multi-antibody IAC cleanup, some mycotoxins had low recoveries and outliers without the use of isotopic internal standards [31].

Different feed ingredients and feed products have complex matrix components, which can enhance or inhibit the ionization of mycotoxin analytes, thereby affecting the accurate quantification of mycotoxins. The stable isotope internal standard has been proven effective to correct matrix effects [32,33]. When the isotope internal standard is used in mycotoxin analysis, the ratio of the isotope internal standard and analyte in feed matrices is stable even after tedious sample preparation because of their nearly identical physical and chemical properties. In other words, the changes of mycotoxins and their isotopic internal standards in feed and feedstuff are synchronized, and losses or promotion of the mycotoxin are completely compensated for by identical losses or promotion of the isotope internal standard [34]. Overall, the use of stable isotope internal standard can better eliminate matrix effects of different feed and feedstuffs, which can effectively improve quantitative accuracy.

However, to the best of our knowledge, there have not been any LC-MS/MS method reports concerning simultaneous determination of the six major mycotoxins in feed using both IAC and stable isotope internal standard. Obviously, it is essential to develop an effective method for monitoring multiple mycotoxins in feed for risk assessment. Hence, the aims of this study were to prepare a novel multi-IAC based on six major mycotoxin antibodies and to develop a sensitive and reliable LC-MS/MS method based on immunoaffinty cleanup and isotope dilution for the determination of six major mycotoxins in feed and feedstuff. The developed method was expected to be a useful tool for feed safety monitoring and exposure assessment of mycotoxins.

## 2. Results and Discussion

### 2.1. Preparation of IACs

#### 2.1.1. Column Capacity

Ten milliliters of phosphate buffered solution (PBS, 0.1 M, pH 7.4, containing 5 µg of DON, ZEN, T-2 toxin and FB_1_, and 1 µg of AFB_1_ and OTA) was taken and passed through an IAC to test the capacity of column. After rinsing with 10 mL of pure water and eluting with 3 mL of methanol–acetic acid solution (98:2, *v*/*v*), the eluate was collected and the concentrations of mycotoxins were then detected by LC-MS/MS. The results of the maximum adsorption capacity of the IAC for six major mycotoxins are shown in Table 1. For trace analysis of mycotoxins in feed samples, the prepared IAC is sufficient for targets purification and super high contaminated samples could be diluted before IAC purification. To our knowledge, most investigations of IACs were mainly focused on single mycotoxin and its metabolites [28,29,35,36], or two to three kinds of mycotoxins [30,37,38], while multifunctional IACs for more than three mycotoxins were rarely reported [31]. The current study developed a multi-antibody IAC and could be applied for six major mycotoxins purification in feed and feedstuff with satisfactory capacities.

#### 2.1.2. Specificity

Ten milliliters of PBS (0.1 M, pH 7.4, containing 0.5 µg of DON, ZEN, T-2 toxin and FB_1_, and 0.05 µg of AFB_1_ and OTA) was taken and passed through an IAC, rinsed with 10 mL pure water, and eluted with 3 mL of methanol–acetic acid solution (98:2, *v*/*v*), and then the eluate was collected for LC-MS/MS analysis to determine the retention of these mycotoxins on the IAC for investigating the specific adsorption of the IAC. The recovery results of six major mycotoxins and their analogs on the IAC are shown in Table 2; all six analytes had recoveries above 95.8%. Except for the six major mycotoxins, the developed multi-antibody based mycotoxin IAC also showed a high adsorption capacity for other analogs of the six mycotoxins with recoveries all above 93.6%, while the other mycotoxins such as citrinin and patulin were not recovered (Table S1). Thus, it has the potential for the purification of the six types of mycotoxins.

### 2.2. Method Optimization

#### 2.2.1. LC-MS/MS Conditions

**Figure 1:**
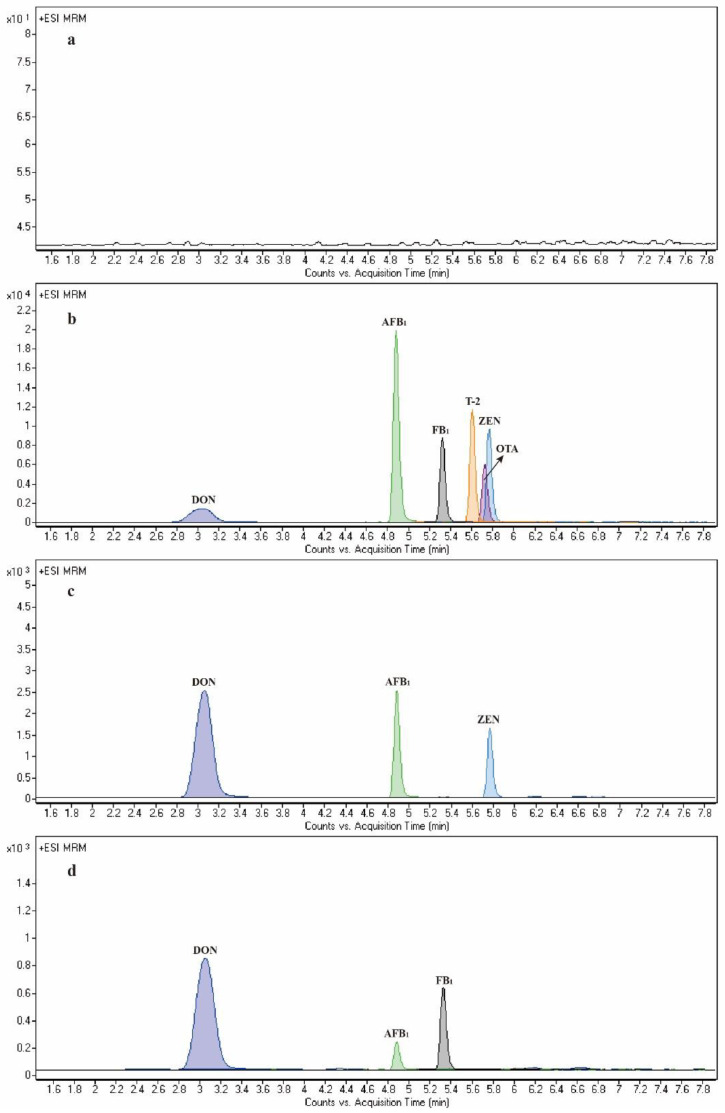
MRM chromatograms for mycotoxin solutions (**a**): blank solvent; (**b**): six major mycotoxins standard solutions, the concentrations were 250, 25, 125, 50, 50 and 500 ng·mL^−1^ for DON, AFB_1_, ZEN, OTA, T-2 toxin and FB_1_, respectively; (**c**): corn sample, DON (929.13 µg·kg^−1^), AFB_1_ (6.79 µg·kg^−1^) and ZEN (71.89 µg·kg^−1^) were detected; (**d**): pig compound feed sample, DON (432.85 µg·kg^−1^), AFB_1_ (2.03 µg·kg^−1^) and FB_1_ (123.32 µg·kg^−1^) were detected).

HPLC was performed by an Acquity UPLC^®^ BEH C18 column under a gradient elution program for analytes separation. Comparing different column temperatures of the chromatographic column, it was found that when the column temperature was lower than 50 °C, the column pressure of the chromatographic column was high and may exceed the pressure limit of the machine, and when the column temperature was 50 °C, the column pressure decreased and became stable. Besides, the quality of each mycotoxin chromatographic peak achieved the best at the flow rate of 0.3 mL·min^−1^. The mobile phase of the six major mycotoxins detected by LC-MS/MS was mostly a methanol–water system, and 0.3% formic acid and 5 mM ammonium formate were reported to be added into the aqueous phase to benefit the ionization efficiency [39]. In this research, addition of 0.15% formic acid and 10 mM ammonium formate increased the ionization efficiency and remarkably improved the chromatographic peak shapes. Finally, a gradient elution procedure described in Section 4.5 was employed for the HPLC mobile phase to obtain good separations and high S/N ratios. The whole HPLC program ran within 10 min, and satisfactory separation and peak shape of most mycotoxins were obtained (Figure 1). Compared with some previous studies for LC-MS/MS simultaneous analysis of mycotoxins, such as the 14 min analysis of DON and ZEN in soil matrix [40], and the 10 min determination of AFB_1_, T-2 toxin, OTA and DON in dried seafood products [41], the developed method has the advantages of shorter analysis time and more detected mycotoxin types.

#### 2.2.2. The Application of Isotope Internal Standards

Effects of the use of isotope internal standards or not were compared based on the recovery of the six mycotoxins in pig compound feed with a certain spiked level and three replicates (*n* = 3) to achieve method optimization. The compared recovery results are shown in Table 3, when isotope internal standards were not used, the calculated recoveries of six major mycotoxins ranged from 47.0% to 109.1% with the relative standard deviations (RSDs) ranging from 4.5% to 24.1%. In particular, the recovery rate of T-2 toxin was as low as 47.0%. Besides, AFB_1_ showed a recovery of 58.1% and a RSD of 24.1%, which indicated a poor analytical accuracy of this method. Actually, there were even several outliers when making the standard curve absence of the isotope internal standard, which affected the accurate quantification. Conversely, higher recoveries of 92.5% to 111.0% and lower RSDs of 0.9% to 8.5% for six mycotoxins in spiked pig compound feed were obtained when isotopic internal standards were used. Finally, isotopic internal standards were performed in this study for correcting matrix effects to achieve satisfactory recoveries and RSDs.

#### 2.2.3. The Selection of Product Ions

**Figure 2:**
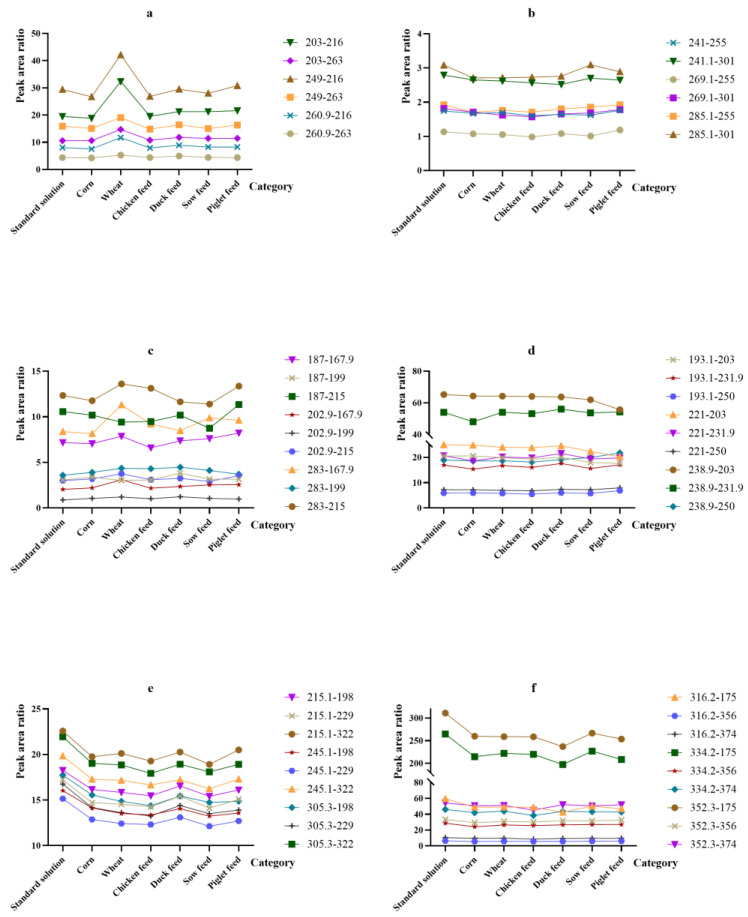
Changes in peak area ratios of different mycotoxins in the standard solution and six feed matrices ((**a**) DON; (**b**) AFB_1_; (**c**) ZEN; (**d**) OTA; (**e**) T-2 toxin; (**f**) FB_1_). Lines with different colors represent the response on the different combination of the selected product ions of the mycotoxin and the isotope internal standard in different feed matrices, and the legends show specific information of product ions combination.

The selection of different product ions will lead to differences in the peak area ratio of mycotoxins and their isotope internal standards between standard solutions and feed samples, thus affecting the accuracy of the results. Whether the peak area ratio of high-response mycotoxins and their isotope internal standard product ions in the standard solutions and different feed matrices is consistent remains to be investigated. Therefore, we screened 2–3 of product ions for each mycotoxin and its isotope internal standard. A certain concentration of standard mycotoxins (half the limit of mycotoxins in pig compound feed set by Chinese government, i.e., 5, 500, 125, 50, 250 and 2500 μg·kg^−1^ for AFB_1_, DON, ZEN, OTA, T-2 toxin and FB_1_, respectively) was then added to six different blank feed matrices including corn, wheat, chicken feed, duck feed, sow feed and piglet feed, and good peak shapes and response values of mycotoxins in different matrices could be obtained under this concentration. In this way, the changes of the peak area ratios of mycotoxins and their isotope internal standards with different product ions in standard products and different feed matrices were investigated. The information of screened product ions is shown in Table 4, and the changes of the peak ratios of different mycotoxins in standard solution and six feed matrices are shown in Figure 2.

In this study, product ions were selected based on the comprehensive consideration of the peak area ratio RSDs and the ion response intensity of mycotoxins in different matrices. For example, when we selected the product ion of “*m*/*z* 285.1” for AFB_1_, the ion response intensity was stronger than that of “*m*/*z* 241”; and when the product ion of “*m*/*z* 241” was selected, the RSD of the peak area ratio of AFB_1_ and ^13^C_17_-AFB_1_ in the six feed matrices and the standard solution was the lowest. The optimal product ions for the six major mycotoxins were finally determined in this way. To our knowledge, it was proposed for the first time that the response of the same product ion in standard solutions and different feed matrices have a certain difference in this study. The detection contents of mycotoxins may more accurate by detecting the response changes of different product ions to various types of feed matrices and screening out a more general product ion.

### 2.3. Method Validation

#### 2.3.1. Linearity and Sensitivity

Linearity was tested by preparing standard curves of the six major mycotoxins. 4 µL of ^13^C_15_-DON, ^13^C_18_-ZEN, ^12^C_24_-T-2 toxin and ^13^C_34_-FB_1_, 2 µL of ^13^C_17_-AFB_1_ and 10 µL of ^13^C_20_-OTA standard solutions were added to 1 mL of each mixed mycotoxin working solution of different concentrations, and the actual concentrations of ^13^C_15_-DON, ^13^C_18_-ZEN, ^12^C_24_-T-2 toxin and ^13^C_34_-FB_1_ were 100 ng·mL^−1^, ^13^C_17_-AFB_1_ 2 ng·mL^−1^, and ^13^C_20_-OTA 10 ng·mL^−1^. The concentrations of each mycotoxin were 5–1000 ng·mL^−1^ for DON, 0.25–50 ng·mL^−1^ for AFB_1_, 2.5–500 ng·mL^−1^ for ZEN, 0.5–100 ng·mL^−1^ for OTA, 2.5–500 ng·mL^−1^ for T-2 toxin and 25–5000 ng·mL^−1^ for FB_1_, respectively. Assays were performed from low to high concentrations. Standard curves were drawn with the peak area ratio of mycotoxins and their isotope internal standards as the ordinate and the mycotoxin concentrations as the abscissa. Standard curve regression equations with acceptable linear relationships (R^2^ over 0.99 for each mycotoxin) were obtained (Table 5).

Sensitivity of this method was assessed by measuring the limit of detection (LOD) and limit of quantification (LOQ) in this study. Limit of detection is monitored on the basis of S/N > 3, and it is described as the lowest detection concentration of targets in samples, while the limit of quantitation is based on S/N > 10 [30]. LODs for targets were 0.075–1.5 µg·kg^−1^ and LOQs were 0.5–5 µg·kg^−1^ in this study (Table 5). The LODs and LOQs of this method were more sensitive than those of the LC-MS/MS method reported in the previous studies [30,42,43], and it can meet the requirements of low-level mycotoxin analysis.

#### 2.3.2. Recovery and Precision

The recoveries, standard deviations (SDs) and RSDs of the six major mycotoxins in four blank feed matrices (corn, wheat, pig feed and chicken feed) with three spike levels and three replicates (*n* = 3) are shown in Table 6. The mean recoveries of the six major mycotoxins in four different feed matrices at three spike levels were 84.2–117.1% with RSDs ranging from 0.2% to 11.6%. The recovery results in our study were similar to the results that the mean recoveries of six zearalenones varied between 82.5% and 106.4% for animal feed sample LC-MS/MS [44] and the mean recoveries of OTA ranged from 82.0% to 109.0% for poultry tissues and eggs sample LC-MS/MS [45]. Besides, compared with the LC-MS/MS method for the simultaneous detection of 15 mycotoxins in aquaculture feed developed by Albero et al. [46], our study showed a better FB_1_ recovery of 94.8–117.1% than that of 10–25%. This results indicated that the established method is reliable, sensitive and suitable for the determination of six major mycotoxins in feed and feedstuff.

#### 2.3.3. Stability

**Figure 3:**
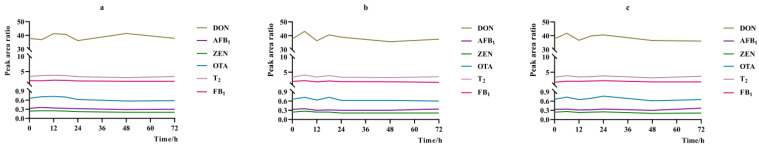
Changes in peak area ratios of six major mycotoxins and their isotope internal standards in pig compound feed with time at different temperatures ((**a**) 4 °C, RSDs all below 9.5% within 72 h; (**b**) 25 °C, RSDs all below 9.7% within 72 h; (**c**) 37 °C, RSDs all below 9.0% within 72 h).

The intermediate concentration in the recovery experiment was selected for the stability studies, and the isotope internal standard was added equally in the standard solutions and feed matrices. In this research, the sample solutions were analyzed at different time points (0, 6, 12, 18, 24, 48 and 72 h) at 4 °C, 25 °C, and 37 °C, respectively. The figures were plotted with the measurement time on the x-axis and the peak area ratio of mycotoxins and their isotope internal standards on the y-axis. The stability results are shown in Figure 3 and Figures S1–S4. The determination results of the six mycotoxins in the standard solution and four feed matrices were stable within 72 h under different temperature conditions, and the RSDs were all less than 9.9%.

### 2.4. Application to Feed Samples for Mycotoxins Analysis

In order to test the reliability of this method, we used the established method to determine the concentrations of six mycotoxins in 36 feed samples including eight corn samples and six wheat samples collected from local feed wholesale market in China, and eight pig compound feed samples, eight chicken compound feed samples and six fermented cattle feed samples from different feed production companies in China. The detection results are shown in Table 7, with more than one mycotoxin detected in all feed samples. The actual sample detection results preliminarily confirmed the general applicability of the established LC-MS/MS method among those common feed samples, and revealed the co-occurrence of multiple mycotoxins in a way. In fact, feed and feedstuffs can be easily contaminated with mycotoxins, and the co-occurrence of mycotoxins is extremely frequent. Franco et al. [47] confirmed a ratio of 51% for the co-occurrence of two or more mycotoxins in 45 maize-based feed samples collected from Brazilian farms. Streit et al. [48] found 38% of the samples were co-contaminated by multiple mycotoxins when investigating the contamination of 17,316 samples of feed and feed raw materials from all over the world. Similarly, a study carried by Arroyo-Manzanares et al. [49] showed that 40% of 228 pig feed samples from Spain were contaminated with more than five mycotoxins. Besides, it is astonishing that all of the 120 pelleted poultry feed samples from Argentina were co-contaminated by FB_1_, HT-2 and T-2 toxin in the research of Monge et al. [50]. It is clear that mycotoxin co-contamination usually raises public concerns because the combination of multiple mycotoxins may result in an additive or synergistic toxicological effects when compared with a single mycotoxin exposure [51].

Indeed, all detected mycotoxin contents among 36 actual feed samples in this study did not exceeding the maximum permitted levels set by the Chinese government (1–5 mg·kg^−1^ for DON, 10–50 µg·kg^−1^ for AFB_1_, 0.15–0.5 mg·kg^−1^ for ZEN, 100 µg·kg^−1^ for OTA, 0.5 mg·kg^−1^ for T-2 toxin and 5–60 mg·kg^−1^ for Fumonisin (B_1_ + B_2_)) [17]. Nonetheless, such feed samples may increase the health risk of long-term feeding for animals due to the co-contamination of multiple mycotoxins. A negative influence of feed conversion was observed in a longitudinal study when broiler chickens were chronically fed a naturally contaminated diet containing low doses of multiple mycotoxins below EU regulatory limits [52]. Additionally, Jia et al. [53] found that a combined dose of DON and ZEN around China’s regulatory limits negatively affected body weight gain and feed consumption and even impaired intestinal functions of piglets. It is necessary to continuously monitor the contamination of mycotoxins in feed and feedstuff and evaluate the impact of co-contamination of low-level mycotoxins on livestock and poultry health. Moreover, similar to the food matrix, more than one contaminant may be present in the same feed. The co-occurrence of mycotoxins and other contaminants in feed such as pesticide and veterinary drug residues, heavy metals and biogenic amines may cause an increased toxicity, and it cannot be ignored [54,55]. Based on the developed LC-MS/MS method of our study, the co-detection technology of mycotoxins and other contaminants in feed can be further explored to comprehensively ensure the quality and safety of feed.

Overall, it is confirmed that the established method can be used for multiple mycotoxin monitoring in feed and feedstuffs. This method achieves a single 10 min run for simultaneous analysis of the six major mycotoxins, which greatly improves the detection efficiency and reduces the running cost of mass spectrometry. Additionally, it shows an outstanding greenness property using less organic solvents compared with conventional liquid–liquid extraction [56]. However, there are still some limitations for this method. Firstly, the detection cost of this LC-MS/MS method is higher than that of the rapid detection method. Secondly, the operation of mass spectrometer is especially complicated, and professional and technical personnel are required for sample analysis. Thirdly, the expensive machine determines that this method is not suitable for on-site detection at the grassroots level.

## 3. Conclusions

In this study, a liquid chromatography-tandem mass spectrometry method based on multi-antibody IAC cleanup and isotope dilution for the analysis of six major mycotoxins in feed and feedstuff was developed. The established LC-MS/MS method had the advantages of good sensitivity, high precision, excellent recoveries, and simple pretreatment operation, which can simultaneously detect DON, AFB_1_, ZEN, OTA, T-2 toxin, and FB_1_ in feed samples. Five types of actual feed samples (total of 36) were detected for the concentrations of the six major mycotoxins using the established method and at least one mycotoxin was detected in all samples, which indicated that the established LC-MS/MS method has strong applicability and can be used for the detection of major mycotoxins in different types of feed samples. In conclusion, this study provides a reliable detection technology for the rapid and simultaneous detection of six major mycotoxins in feed and feedstuff.

## 4. Material and Methods

### 4.1. Chemicals and Reagents

1 milligram standard mycotoxins powders of AFB_1_, ZEN, DON, OTA, T-2 toxin and FB_1_ (subsequently dissolved in acetonitrile to give solutions of 1 mg·mL^−1^ and stored at −20 °C), 1 mL isotope internal standard solutions of ^13^C_17_-AFB_1_, ^13^C_18_-ZEN, ^13^C_15_-DON, ^13^C_20_-OTA, ^12^C_24_-T-2 toxin and ^13^C_34_-FB_1_ prepared in acetonitrile and stored at −20 °C (25 µg·mL^−1^ for ^13^C_15_-DON, ^13^C_18_-ZEN, ^12^C_24_-T-2 toxin and ^13^C_34_-FB_1_, 0.5 µg·mL^−1^ for ^13^C_17_-AFB_1_ and 1 µg·mL^−1^ for ^13^C_20_-OTA), and Sephrose 4B gel (CNBr-activated) for IAC preparation were all purchased from Sigma-Aldrich (St. Louis, MO, USA). Acetonitrile, methanol, formic acid and ammonium formate (HPLC grade) were also from Sigma-Aldrich (St. Louis, MO, USA). Hydrochloric acid (HCl), sodium bicarbonate (NaHCO_3_), sodium chloride (NaCl), tris, glacial acetic acid, potassium chloride, disodium hydrogen phosphate, potassium dihydrogen phosphate, sodium dihydrogen phosphate, and sodium azide (NaN_3_) were of analytical grade, purchased from Beijing Chemical Reagent Company (Beijing, China). A Milli-Q water purification system was obtained from Millipore (Bedford, MA, USA), and the resistivity of water was 18.2 MΩ·cm at 25 °C.

### 4.2. Apparatus

High performance liquid chromatograph and Tandem quadrupole mass spectrometer were purchased from Agilent Technologies (Santa Clara, CA, USA). Electronic analytical balance (BSA124S) was from Sartorius (Beijing, China). Sartolab^®^ RF filter was from Sartorius (Shanghai, China). Vortex mixer (Model HQ-60) was obtained from North TZ-Biotech. Co., Ltd. (Beijing, China). High-speed cryogenic centrifuge (Biofilge 22R) was purchased from Heraeus (Hanau, Germany), and nitrogen evaporator (HSC-24B) was purchased from Beijing Chenxi Yongchuang Technology Co., Ltd. (Beijing, China).

### 4.3. Preparation of IACs

#### 4.3.1. Matrix Preparation

An amount of 3 g (±0.02 g) of base powder (CNBr-activated Sephrose 4B) was weighed and dissolved in 10 mL of 1 mM HCl. The matrix swelled immediately and then was placed in a sintered glass filter (porosity: G3) and washed with 1 mM HCl for 15 min. Approximately 400 mL of 1 mM HCl were used in portions.

#### 4.3.2. Ligand Conjugation

Briefly, the swollen CNBr-activated Sepharose 4B was washed with 10 mL of coupling buffer (0.5 M NaCl, 0.1 M NaHCO_3_, pH 8.3) and quickly transferred into the antibody solution. Then, 3 g of matrix Sepharose 4B was conjugated with 45 mg of mycotoxins monoclonal antibodies (3 mg of AFB_1_ antibody, 3 mg of OTA antibody, 6 mg of ZEN antibody, 10 mg of DON antibody, 15 mg of FB_1_ antibody and 8 mg of T-2 antibody, the antibody mixture was optimized based on the expected capacity of IAC by trial and error). Next, the above mixture was fully mixed in an end-over-end manner under room temperature (20–25 °C) for 2 h, or at 4 °C overnight. Then, the mixture was centrifuged at 4 °C, 376× *g* for 1 min, and the supernatant was transferred to a new centrifuge tube and the OD_280_ nm value was measured. In the next step, the Sepharose 4B at the bottom of the centrifuge tube was taken and washed with at least 5 times the volume of matrix (gel) coupling buffer to remove excess ligand. Then the matrix was transferred to 0.1 M Tris-HCl buffer (pH 8.0) or 1 M ethanolamine (pH 8.0) for 2 h at room temperature or 16 h at 4 °C to block all remaining active groups. In order to remove the excess ligands that were not coupled after coupling, the matrix was washed with low and high pH buffers in sequence at least 5 times the volume of each matrix for at least 3 cycles.

#### 4.3.3. Packing in Columns

In the research, wet packing was used. After the column was packed, 5 times the column bed volume of 0.01% NaN_3_-PBS (sterile filtered by Sartolab^®^ RF filter) was passed through the column, and 0.01% NaN_3_-PBS was used for storage.

### 4.4. Sample Preparation

An amount of 5 g (±0.02 g) of feed samples was weighed and transferred to a 50 mL polypropylene centrifuge tube, then 1 g of sodium chloride was added. Later, 20 mL of acetonitrile–water mixture (60:40, *v*/*v*) was added to samples and vortexed for 30 min for extraction. Then, each sample was centrifuged at 4 °C, 6010× *g* for 10 min, and the supernatant was filtered into another 50 mL tube. Next, 2.0 mL of supernatant was diluted with 48.0 mL of 1% Tween-20 in PBS (PBST, pH 7.4). Then, 20 mL of the diluted supernatant was taken and 4 µL of ^13^C_15_-DON, ^13^C_18_-ZEN, ^12^C_24_-T-2 toxin and ^13^C_34_-FB_1_, 2 µL of ^13^C_17_-AFB_1_ and 10 µL of ^13^C_20_-OTA standard solutions were added for use.

The 20 mL filtrate in the above step was all passed through the IAC at a flow rate of 1–2 drops per second until air entered the IAC. Next, 10 mL of PBS (0.1 M, pH 7.4) was passed through the IAC at a flow rate of 1–2 drops per second until air entered the IAC. Then, the IAC was rinsed with 3 mL of methanol–acetic acid solution (98:2, *v*/*v*) at a flow rate of 1 drop per second, and the eluent was collected in a glass test tube. After concentrated and dried under nitrogen at 50 °C, the volume was made up to 1 mL with methanol–water (50:50, *v*/*v*) for LC-MS/MS analysis.

### 4.5. LC-MS/MS Analysis

Chromatographic separation for six major mycotoxins was performed on an Acquity UPLC^®^ BEH C18 Column (1.7 μm, 2.1 × 100 mm) with column temperature at 50 °C. Mobile phase A (methanol, 0.05% formic acid) and mobile phase B (water, 0.15% formic acid, 10 mM ammonium formate) were used. Gradient elution program was used with initial mobile phase at 15% of Solvent A and 85% of solvent B. From 0 to 0.5 min, solvent A maintained at 15%; 0.5–4 min, solvent A increased to 100%; 4–7 min, solvent A maintained at 100%; 7–7.1 min, solvent A decreased to 15%; 7.1–10 min, and solvent A maintained at 15%. The injection volume was 10 μL and follow rate was 0.3 mL·min^−1^.

The mass spectrometry was run with electrospray ion source and all the mycotoxins were detected in positive mode with other MS parameters as follows: the capillary voltage was set at 3500 V; drying gas temperature, 350 °C; drying gas flow, 5 L·min^−1^; Nebulizer, 50 psi; sheath gas temperature, 350 °C; sheath gas flow, 7 L·min^−1^. MS detection was performed in multi reaction monitoring mode (MRM, parameters are shown in Table 8).

### 4.6. Result Calculation

The mass fraction of the six mycotoxins in the feed samples was calculated according to the following formula:

(1) $$\omega =\frac{c\times V_{0}\times V_{2}\times V_{4}}{m\times V_{1}\times V_{3}}$$

where:

*ω* was the mass fraction of mycotoxins (AFB_1_, ZEN, DON, OTA, T-2 toxin and FB_1_) in the sample (μg·kg^−1^);

*c* was the concentration of mycotoxins in the sample solution obtained from the standard curve (ng·mL^−1^);

*V*_0_ was the volume of the extraction solution (mL);

*V*_1_ was the volume of the supernatant taken after extraction and centrifugation (mL);

*V*_2_ was the total volume of the solution after dilution with PBST (mL);

*V*_3_ was the volume of diluted solution purified by IAC (mL);

*V*_4_ was the reconstituted volume after nitrogen blowing (mL);

*m* was the weight of the sample (g).

All data used for statistical material can be found in the Supplementary Material Table S1 and Figures S1–S4.

## Figures and Tables

**Table 1:** The maximum adsorption capacity of the IAC for six major mycotoxins.

Mycotoxins	Column Capacity (ng)
DON	995
AFB_1_	198
ZEN	998
OTA	100
T-2 toxin	996
FB_1_	2995

**Table 2:** Recoveries of six major mycotoxins on the IAC.

Mycotoxins	Recovery (%)
DON	97.9
AFB_1_	100.0
ZEN	99.5
OTA	99.8
T-2 toxin	98.9
FB_1_	95.8

**Table 3:** Comparison about recovery results of mycotoxins in pig feed with or without the use of isotope internal standards (*n* = 3).

Analyte	Spiked Level (μg·kg^−1^)	Without Isotope Internal Standards		With Isotope Internal Standards	
		Mean Recovery (%)	RSD (%)	Mean Recovery (%)	RSD (%)
DON	1000	74.8	4.5	96.0	3.9
AFB_1_	10	56.1	24.1	93.7	6.8
ZEN	250	78.1	16.2	103.7	0.9
OTA	100	109.1	6.3	92.5	8.5
T-2 toxin	500	47.0	11.8	99.7	2.4
FB_1_	5000	72.3	20.7	111.0	6.2

**Table 4:** Product ion screening of mycotoxins and their isotope internal standards.

Mycotoxins	Product Ions (*m*/*z*)	Internal Standards	Product Ions (*m*/*z*)
DON	203/249/260.9	^13^C_15_-DON	216/263
AFB_1_	241.0/269.1/285.0	^13^C_17_-AFB_1_	255/301
ZEN	187/202.9/283	^13^C_18_-ZEN	167.9/199/215
OTA	193.1/221/238.9	^13^C_20_-OTA	203/231.9/250
T-2 toxin	215.1/245.1/305.3	^12^C_24_-T-2	198/229/322
FB_1_	316.2/334.2/352.3	^13^C_34_-FB_1_	175/356/374

**Table 5:** Parameters of standard curves, LOD, and LOQ for mycotoxins.

Analyte	Liner Range (µg·kg^−1^)	Standard Curve	R^2^	LOD (µg·kg^−1^)	LOQ (µg·kg^−1^)
DON	5–1000	y = 0.0133x − 0.0903	0.9983	0.75	2.5
AFB_1_	0.25–50	y = 0.0567x − 0.0119	0.9954	0.075	0.25
ZEN	2.5–500	y = 0.0183x + 0.0439	0.9996	0.375	1.25
OTA	0.5–100	y = 0.1075x − 0.0944	0.9983	0.15	0.5
T-2 toxin	2.5–500	y = 0.0100x − 0.0189	0.9990	0.15	0.5
FB_1_	25–5000	y = 0.0070x + 0.0835	0.9983	1.5	5

**Table 6:** Recovery and precision of mycotoxins in different feed matrices (*n* = 3).

Analyte	Spike Level (μg·kg^−1^)	Matrix	Mean Recovery (%)	SD (μg·kg^−1^)	RSD (%)
DON	500, 1000, 2000	Corn	94.9–100.8	6.4–49.0	1.3–5.2
		Wheat	94.1–97.7	9.4–59.2	1.7–6.3
		Pig feed	93.9–99.4	7.6–92.7	1.5–4.9
		Chicken feed	96.1–103.6	27.3–61.6	3.0–5.4
AFB_1_	5, 10, 20	Corn	101.0–114.8	0.4–1.1	4.6–6.7
		Wheat	105.3–111.3	0.2–0.5	1.2–10.1
		Pig feed	93.7–105.0	0.1–1.7	2.5–8.9
		Chicken feed	84.2–104.3	0.4–1.9	6.5–10.3
ZEN	125, 250, 500	Corn	103.2–109.0	3.6–14.5	2.7–3.9
		Wheat	94.9–108.5	2.6–13.3	0.9–10.7
		Pig feed	102.0–106.0	2.3–18.4	0.9–11.2
		Chicken feed	97.6–116.5	10.4–13.7	2.4–8.4
OTA	50, 100, 200	Corn	96.3–110.3	1.1–7.0	2.1–3.6
		Wheat	98.0–111.8	2.7–6.0	1.6–10.7
		Pig feed	92.5–101.8	1.3–12.4	2.7–6.1
		Chicken feed	100.8–113.7	3.2–8.6	3.8–5.7
T-2 toxin	250, 500, 1000	Corn	92.0–100.7	2.6–13.4	1.1–1.7
		Wheat	89.4–103.9	1.3–22.3	0.3–2.3
		Pig feed	89.3–99.7	3.3–16.5	1.5–2.4
		Chicken feed	95.4–104.4	0.9–15.2	0.2–4.2
FB_1_	2500, 5000, 10,000	Corn	106.1–113.3	144.2–340.6	3.0–8.9
		Wheat	95.5–106.4	125.0–277.0	1.4–11.6
		Pig feed	95.7–117.1	247.5–485.0	5.1–11.4
		Chicken feed	94.8–116.6	240.3–285.8	2.4–10.1

**Table 7:** Analysis of mycotoxins detection results of feed samples.

Analyte	Feed samples	Corn	Wheat	Pig Compound Feed	Chicken Compound Feed	Fermented Cattle Feed
	Number of Samples	8	6	8	8	6
DON	Detectable samples ^**a**^	8	4	8	8	6
	Detection rate (%)	100	66.7	100	100	100
	Content range (μg·kg^−1^)	339.50–1403.22	65.83–986.42	47.86–865.23	3.94–727.16	4.98–38.08
AFB_1_	Detectable samples	5	2	2	3	3
	Detection rate (%)	62.5	33.3	25.0	37.5	50.0
	Content range (μg·kg^−1^)	5.64–11.48	4.09–6.79	6.59–11.96	2.03–31.08	0.30–0.63
ZEN	Detectable samples	8	5	8	8	6
	Detection rate (%)	100	83.3	100	100	100
	Content range (μg·kg^−1^)	2.85–208.40	5.10–71.89	10.14–284.45	10.15–228.58	20.40–149.33
OTA	Detectable samples	6	3	2	2	3
	Detection rate (%)	75.0	50.0	25.0	25.0	50.0
	Content range (μg·kg^−1^)	4.60–15.05	1.63–11.66	6.54–8.40	1.97–6.54	1.82–2.79
T-2	Detectable samples	5	3	5	4	0
	Detection rate (%)	62.5	50.0	62.5	50.0	0
	Content range (μg·kg^−1^)	2.42–547.61	2.64–302.36	0.3–402.78	1.93–87.20	–
FB_1_	Detectable samples	2	2	7	8	6
	Detection rate (%)	25.0	33.3	87.5	100	100
	Content range (μg·kg^−1^)	6.02–680.93	21.90–709.00	33.55–147.11	15.19–2013.44	78.00–6220.95

^a^ Sample with concentration > LOQ.

**Table 8:** MS/MS parameters of six major mycotoxins and their isotope internal standards in MRM mode.

Mycotoxins	Type	Precursor Ions (*m*/*z*)	Product Ions (*m*/*z*)	Retention Time (min)	Fragmentor (V)	Collision Energy (eV)
DON	[M+H]^+^	297.1	249 *	3.067	110	10
			203			6
AFB_1_	[M+H]^+^	313.1	241 *	4.884	130	38
			285			24
ZEN	[M+H]^+^	319.1	283 *	5.767	80	8
			187			20
OTA	[M+H]^+^	404.1	238.9 *	5.724	90	21
			221			15
T-2 toxin	[M+H]^+^	484.2	215.1 *	5.606	80	15
			305.3			8
FB_1_	[M+H]^+^	722.4	352.3 *	5.322	135	36
			334.2			44
^13^C_15_-DON	[M+H]^+^	312.2	263 *	3.090	110	8
			216			14
^13^C_17_-AFB_1_	[M+H]^+^	330.1	255 *	4.882	145	40
			301			30
^13^C_18_-ZEN	[M+H]^+^	337.1	199 *	5.765	80	20
			167.9			40
^13^C_20_-OTA	[M+H]^+^	424.1	250 *	5.725	90	26
			231.9			40
^12^C_24_-T-2	[M+H]^+^	508.2	229 *	5.605	80	13
			322			9
^13^C_34_-FB_1_	[M+H]^+^	756.4	356 *	5.322	135	45
			374			50

* Quantitative ion.

## Data Availability

Not applicable.

## Supplementary Materials

The following supporting information can be downloaded at: https://www.mdpi.com/article/10.3390/toxins14090631/s1, Table S1: Recoveries of other mycotoxins on the IAC; Figure S1: Changes in peak area ratios of six major mycotoxins and their isotope internal standards in standard solutions with time at different temperatures (a. 4 °C, RSD all below 9.9% within 72 h; b. 25 °C, RSDs all below 8.4% within 72 h; c.37 °C, RSDs all below 9.9% within 72 h); Figure S2: Changes in peak area ratios of six major mycotoxins and their isotope internal standards in corn with time at different temperatures (a. 4 °C, RSDs all below 9.5% within 72 h; b. 25 °C, RSDs all below 7.7% within 72 h; c.37 °C, RSDs all below 9.6% within 72 h); Figure S3: Changes in peak area ratios of six major mycotoxins and their isotope internal standards in wheat with time at different temperatures (a. 4 °C, RSDs all below 9.8% within 72 h; b. 25 °C, RSDs all below 9.8% within 72 h; c.37 °C, RSDs all below 9.9% within 72 h); Figure S4: Changes in peak area ratios of six major mycotoxins and their isotope internal standards in chicken compound feed with time at different temperatures (a. 4 °C, RSDs all below 9.8% within 72 h; b. 25 °C, RSDs all below 9.9% within 72 h; c.37 °C, RSDs all below 9.2% within 72 h).
