# Supplementary material for: Immunoaffinity Cleanup and Isotope Dilution-Based Liquid Chromatography Tandem Mass Spectrometry for the Determination of Six Major Mycotoxins in Feed and Feedstuff
Source: Toxins (Basel). 2022 Sep 11;14(9):631. doi: 10.3390/toxins14090631 (PMC9503004; doi:10.3390/toxins14090631)
Supplement: Supplementary file 1 [file toxins-14-00631-s001.zip › toxins-1886421-supplementary.pdf]

toxins

Article

Immunoaffinity Cleanup and Isotope Dilution-Based Liquid Chromatography Tandem Mass Spectrometry for the Determination of Six Major Mycotoxins in Feed and Feedstuff

Ying Liu, Yongpeng Jin, Qi Guo, Xiong Wang, Sunlin Luo, Wenjun Yang, Juntao Li and Yiqiang Chen

Table S1. Recoveries of other mycotoxins on the IAC.

Mycotoxins	Recovery (%)
3-Acetyl deoxynivalenol	97.8
Aflatoxin B_2	96.8
Aflatoxin G_1	95.6
Aflatoxin G_2	96.3
Aflatoxin M_1	98.6
Aflatoxin M_2	95.2
α-Zearalanol	99.5
β-Zearalanol	95.9
Zearalanone	96.2
α-Zearalenol	97.4
β-Zearalenol	95.4
Ochratoxin B	96.2
Ochratoxin C	97.4
HT-2 toxin	95.9
Fumonisin B_2	94.3
Fumonisin B_3	93.6
Citrinin	0
Patulin	0
Diacetoxyscirpenol	0

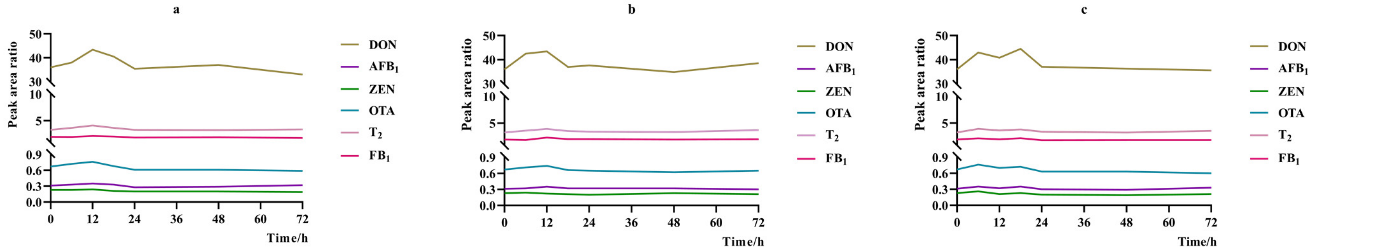

Figure S1.: Changes in peak area ratios of six major mycotoxins and their isotope internal standards in standard solutions with time at different temperatures (a. 4 °C, RSD all below 9.9% within 72 h; b. 25 °C, RSDs all below 8.4% within 72 h; c.37 °C, RSDs all below 9.9% within 72 h).

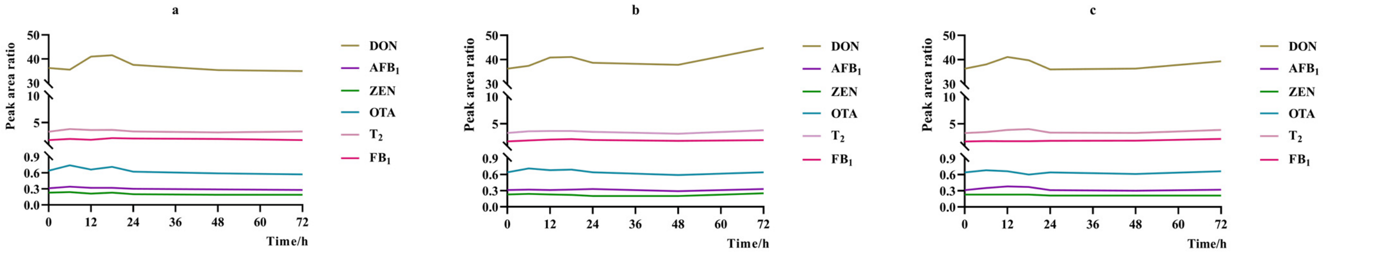

Figure S2.: Changes in peak area ratios of six major mycotoxins and their isotope internal standards in corn with time at different temperatures (a. 4 °C, RSDs all below 9.5% within 72 h; b. 25 °C, RSDs all below 7.7% within 72 h; c.37 °C, RSDs all below 9.6% within 72 h).

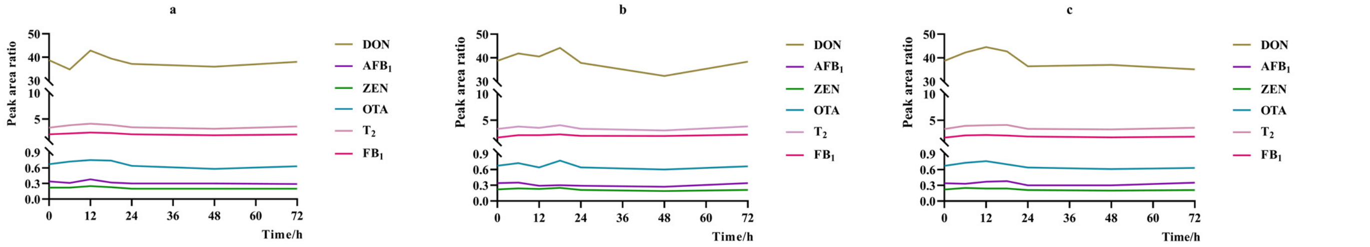

Figure S3. Changes in peak area ratios of six major mycotoxins and their isotope internal standards in wheat with time at different temperatures (a. 4 °C, RSDs all below 9.8% within 72 h; b. 25 °C, RSDs all below 9.8% within 72 h; c.37 °C, RSDs all below 9.9% within 72 h).

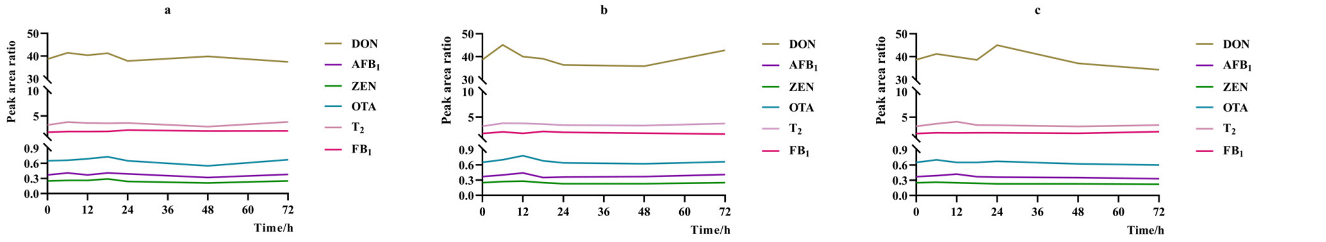

Figure S4. Changes in peak area ratios of six major mycotoxins and their isotope internal standards in chicken compound feed with time at different temperatures (a. 4 °C, RSDs all below 9.8% within 72 h; b. 25 °C, RSDs all below 9.9% within 72 h; c.37 °C, RSDs all below 9.2% within 72 h).
